# Retraction: MicroRNA-377 Suppresses Cell Proliferation and Invasion by Inhibiting TIAM1 Expression in Hepatocellular Carcinoma

**DOI:** 10.1371/journal.pone.0266302

**Published:** 2022-03-24

**Authors:** The PLOS ONE Editors

After this article [1] was published, similarities were noted between this article and submissions by other research groups (including [2]) which call into question the validity and provenance of the reported results and the adherence of this article to the PLOS Authorship policy. It also came to light that there is extensive text overlap between this article [1] and previously published work.

In light of these issues, the *PLOS ONE* Editors retract this article.

The authors either could not be reached or did not respond to recent correspondence about this matter.
